# Supplementary material for: Majority of pediatric dengue virus infections in Kenya do not meet 2009 WHO criteria for dengue diagnosis
Source: PLOS Glob Public Health. 2022 Apr 20;2(4):e0000175. doi: 10.1371/journal.pgph.0000175 (PMC10021889; doi:10.1371/journal.pgph.0000175)
Supplement: Attachment — Submitted filename: Resonse to Reviewers PLOS GPH.docx [file pgph.0000175.s001.docx]

Dear Carlos Ramirez-Soto and the editorial board at PLOS Global Public Health,

We would like to thank you very much for the excellent review of our paper (manuscript ID PGPH-D-21-00576) entitled, “Majority of Pediatric Dengue Virus infections in Kenya Do Not Meet 2009 WHO Criteria For Dengue Diagnosis." We have addressed the reviewer comments and feel that our paper is now suitable for publication to PLOS Global Public Health.

Please find below point-by-point responses to the reviewer comments. The original comments are italicized and responses are regular text. The changes made in the manuscript are now highlighted and noted with tracked changes.

Sincerely,

Aslam Khan, DO

Fellow, Department of Pediatrics, Division of Infectious Diseases

Stanford University School of Medicine

Biomedical Innovation Building, 2 Floor, Room 2400,

1291 Welch Road, Stanford, CA, 94305

Email: akhan1@stanford.edu

*Review Comments to the Author* (Responses below each comment)

*Please use the space provided to explain your answers to the questions above. You may also include additional comments for the author, including concerns about dual publication, research ethics, or publication ethics. (Please upload your review as an attachment if it exceeds 20,000 characters)*

*Reviewer #1: I think it would be important to compare the absolute numbers and
percentages of the findings of this study with respect to the classification criteria of the WHO, with other studies and include them in the discussion. They could also expand the information if the cases were obtained in times of greater or lesser dengue transmission and in the future increase the sample size*

REPLY: Thank you for your response. We cited multiple studies for comparison and included a few reference statistics in the discussion.

*Reviewer #2: the statistical analysis is not complete as the "p-value" and the confidence Interval have not been calculated.*

*- During the analysis of the results, the "p-value" was not calculated, nor the confidence intervals, not allowing to conclude that the results were not due to chance alone (see page 11).*

*-we suggest that in the "Results" section on page 8 and page 11 of the manuscript, the authors may complete the data analysis by calculating the "p-value" and the confidence intervals.*

REPLY: Thank you for your review and response. Our statistics are all largely simple proportions from a single population. There is no comparison group and therefore we cannot calculate a p-value using any appropriate test. We did however calculate 95% confidence intervals by calculating the exact binomial confidence interval. We are not able to produce p-values given the lack of a comparison group as this is all one population.

*- The absence of biological test in the research protocol is a problem knowing that Dengue symptoms are not very specific and that biological criteria are part of the classification of new cases of Dengue, both simple and severe. (see page 7 and Figure 1 page 11 of the manuscript)*

REPLY: Thank you for your response. Unfortunately this study was funded to support the dengue virus and chikungunya virus testing but additional laboratory studies like a complete blood count were not funded and would be at cost to the participants. There were some individuals with laboratory data but it was not complete as most families elected to not undergo additional testing due to the financial burden.

*- The RT-PCR test should also be used to search for other arboviruses besides Chikungunya (Zika virus, Yellow fever, Rift Valley fever, Crimean-Congo virus, etc...) as there are cross-reactions between them which can lead to false positive cases of Dengue. (see page 6).
- If the samples are still available, may the authors do the testing for other arboviruses in order to be sure that the RT-PCR is positive for the Dengue Virus for the cases that not meet the WHO criteria.*

REPLY: Thank you for your response. I have included the references for our RT-PCR testing. There is high specificity with the dengue RT-PCR and low false positive rates with dengue. In general with the high specificity and nucleic acid testing, there is a very low probability of cross-reactivity or misidentification with other arboviruses. This is in contrast to NS1 antigen testing or some serologic assays. We do not feel there is a need to test for other viruses as these were reliably positive for dengue virus with ongoing internal controls and periodic assessments for quality control of the assay.

*Reviewer #3: The paper does not present enough evidence to support its single most overriding communication objective, which is that WHO revised case definition is not sensitive for Dengue fever. The case definition for probable dengue includes two of five symptoms, one of which (leucopenia) was never assessed in all cases. It is surprising however that children numbering as much who are visiting medical facilities including reference hospitals with febrile illness do not have records of blood counts.*

REPLY: Thank you for your response. We do acknowledge this finding as one limitation of the study. We understand individuals do not have entire workup to make a diagnosis. After review of each chart, if every individual did have leukopenia, 74 individuals will still not meet criteria for disease. In review of other studies, reports have demonstrated as high as 66% of individuals presenting with leukopenia on admission with a dengue diagnosis. If applied to this cohort just under 50% of the participants would not meet criteria, supporting the underlying hypothesis. We appreciate this critique from the reviewer and have addressed this point in the text.

*The methods section did not offer a full description of the diagnostic process. For example, no information was given as to the name, description or sources of the primers/kits used in the polymerase chain reaction performed in the field sites and at Stanford University. Such would offer opportunity for appraisal of the quality of these primers and their capacity to prevent cross-reactivity with other febrile illness causing viruses.*

REPLY: Thank you for your response. We have included the references for the RT-PCR testing which provide all information for the primers used and validation of the assay.

*Data as presented in table 1 is not clear and no reference to this table was made in the discussion that can help clarify what figures and percentages represent.*

REPLY: Thank you for this response. We have addressed this point in the text as marked.
